# Racial Capitalism and the Dialectics of Development: Exposing the Limits and Lies of International Economic Law

**DOI:** 10.1007/s10978-022-09336-z

**Published:** 2022-11-26

**Authors:** Mohsen al Attar, Claire Smith

**Affiliations:** 1 School of Law, Faculty of Laws, University of Warwick and Visiting Scholar, UCL, Coventry, UK; 2 School of Law, University of Amsterdam, Amsterdam, Netherlands

**Keywords:** Racial capitalism, international economic law, the Black Radical Tradition, expropriation, exploitation, and the dialectics of development

## Abstract

International economic law is peculiar. It claims universal character, yet eschews engagement with many, if not all, the racialised features of the global political economy. Its scholars mostly ignore imperialism, colonialism, and capitalism; they exclude slavery, predation, and racism altogether. In the following article, we draw upon Walter Rodney’s dialectics of development to offer a racial capitalist critique of international economic law. The disciplinary boundaries and operative logic normalised by its denizens corral us in a white, Eurocentric episteme. Ahistoricism, decontextualisation, and externalisation are three epistemic devices at the forefront of the exclusionary discourse of IEL. In this space, the histories and epistemologies of Black peoples are ghettoised, treated as alien to the framework. After identifying this bias, we use the Black Radical Tradition to evaluate IEL’s amenability to the racial capitalism critique.

## Introduction

The only place where Negroes did not revolt is in the pages of capitalist historians.

CLR James

When we speak of race, we conjure emotions, meanings, and memories; ghosts and premonitions are not far off, either. Social constructs like race live and die in history, shaped by factors too complex to disentangle or disaggregate. Imagination produces value which we reify via the ensuing interactions, converting the contingent into the conventional. As critical legal scholars, we approach race as a theoretical tool to explain aspects of the human condition. By treating race as a lens, we can critique social phenomena like international economic law (IEL) with greater rigour, achieving a deeper understanding of the world we inhabit and the stratifications we navigate.

Scholars and students of IEL rarely engage with questions of race, racialisation, or the resulting racism. These concepts are absent from standard textbooks and journals of IEL. Omitting racialisation, which seizes IELs role in the ongoing social construction of race, is especially jarring, working to reinforce liberal fallacies (Richardson 2001, p. 75). To IEL scholars, formal equality and anti-discrimination are fundamental (Thomas 1999, p. 2). Yet, this shadow version of equality cohabits neatly with market logic, reducing manifestations of inequality to isolated perversions. The critical views that surface target the usual suspects of poverty, political authoritarianism, or social mobility. In standard discourses on IEL, the triumph of liberalism and, increasingly, neoliberalism is absolute (Issar 2021).

Scholars of racial capitalism situate race, exploitation, and inequitable development at the forefront of their analytical frame (Melamed 2015). They expose capitalism as a racially violent order that imposes processes of racialised exploitation and dehumanisation; it does so in the name of white supremacy and mammon (Dawson 2016). Scholars eschew the mythological reconstructions of history proffered by Europe, offering a historical re-remembering that foregrounds the experiences, agency, and humanity of those who suffer the status quo (Rice 2020).^1^ Driven by humanistic ideals, they materially support the flourishing of all peoples.

The racial capitalist critique, however, is incongruent with IEL: revolutionary peg to IEL’s capitalist hole. The disconnect between IEL’s racialising logic and racial capitalism’s subversive ambition feels impossible to reconcile. For example, IEL scholars presume capitalism rather than engage it as a contingent, cultural, and ideological construct. To racial capitalism scholars, the reform of capitalism is impossible. Here lie our reservations about the surge of scholarship on racial capitalism: to explore the possibility of an IEL sympathetic to racialisation and racism risks legitimising the scourge racial capitalism’s critique seeks to eradicate. We thus probe IEL’s epistemic contours, testing the field’s amenability to the critique.

We proceed in three parts. First, we examine the interplay between race and economic exploitation. Next, we link racial capitalism to IEL’s epistemic, normative, and operational biases, revealing the incongruity between the critique and the disciplinary boundaries that corral IEL’s logic. Third, we contemplate the possibility of anti-racist IEL. If the ascendancy of capitalism racialised the world, IEL sustained its longevity. Is it capable of doing otherwise? We link this discussion to the Black Radical Tradition (BRT). As capitalism matured through practices of racial oppression, to proponents of the BRT, an end to racism compels an end to capitalism, obliging us to conclude with the notion of rupture. If we wish to redress the racialised conditions that plague the modern world, we posit the need for a new rupture.^2^ Racial capitalism scholars have written about this inevitability, exploring non-sovereignty and ungovernability as legitimate forms of being. Orthodox IEL scholars will not welcome such rupture, for it would collapse the epistemological presumptions on which IEL and European modernity survive. However, for the Black peoples of the world, a collapse may allow them to breathe freely once again.

## Race, Racism, and Racial Capitalism: A Self-Sustaining Matrix

To win the Negro women for full participation in the antifascist, anti-imperialist coalition, to bring her militancy and participation to even greater heights in the current and future struggles against Wall Street imperialism, progressives must acquire political consciousness as regards her special oppressed status.

Claudia Jones

Proponents of capitalism represent the model in innocuous, theoretical, and aspirational tones (Friedman 2002). Qureshi and Ziegler declare the Bretton Woods institutions are predicated on a ‘market economy and the promotion of global welfare’ (2019, p. 10). In the next sentence, they assert the theory of comparative advantage increases ‘global economic welfare’ (Qureshi and Ziegler 2019, p. 11). Quoting the Charter of Paris for a New Europe, Herdegen is equally effusive: ‘Freedom and political pluralism are necessary elements in our common objective of developing market economies towards sustainable economic growth, prosperity, social justice, expanding employment and efficient use of economic resources’ (2016, p. 159). The stakes are high for ‘the success of the transition to a market economy by countries making efforts to this effect is important and in the interest of us all,’ Herdegen maintains, as it ‘will enable us to share a higher level of prosperity which is our common objective’ (2016, p. 159). Capitalism and market economies, terms used interchangeably, are engines of productivity, innovation, and welfare, stimulating subsequent stages of human development.

Yet, gazing beyond the hagiographical haze, we uncover capitalism’s machinery and morality. As both Morgan (2021) and Bhandar (2017) describe in gruelling detail, capitalism’s parameters shape a specific social order, one that demands the patterns of racialisation we deplore. Each underscores the knitting of capitalist precepts within social relations, depicting an economic model that has gained existential character. To capitalists, we must accumulate for accumulation’s sake, and the impossibility of this ahistorical aspiration is inconsequential (Strauss 2008). Yet, when the postulate of endless accumulation collides with the materiality of scarcity, we square up against the dialectics of development. Domination and unequal distribution are natural outgrowths of a capitalist order operating in a universe of scarce resources, making it obvious why capitalism’s interlocutors prefer the realms of theory and modelling. In these fabricated spaces, capitalism facilitates the constitution and distribution of private property rights via efficient market mechanisms. And it does so in the name of *shared prosperity, social justice, and global welfare*.

It is here we begin our investigation into the racialised structures of IEL. Scholars of racial capitalism recognise that capitalism is also a political system that allocates influence and authority to the wealthiest subjects. It is plutocratic not democratic, unmasking the inequality that undergirds a liberal market economy. As Amin conveys:

That group plutocracy dominates the current globalization which it has indeed itself shaped (not to say ‘constructed’) to suit its sole narrow interests. It has substituted for the ancient ‘international (unequal) division of labor,’ based on the centers/peripheries contrast, a financial geography, i.e., an integration of transnational ‘territories.’ This geography is the product of the strategies of the groups under consideration, and not a ‘reality’ external to it. It shapes in turn what appears as ‘international trade’ and what has become in reality and in growing proportions a transfer of wealth on behalf of certain plutocratic groups. (2008, p. 58)

It is impossible to understand IEL without reflecting on the inequality inherent to capitalism, yet IEL scholars are cagey about the topic. It does not feature in the index or substantive text of the IEL coursebooks surveyed (Qureshi and Ziegler 2019; Herdegen 2016; Shaw 2021; Evans 2018), except as an after-thought on page 729 of Choukroune and Nedumpara’s text (2022). The Journal of International Economic Law’s (JIEL) record is consistent, a powerful indictment considering its centrality in circulating scholarship on IEL and recent foray into racial capitalism. Using Heinonline’s search function, we uncover references to ‘capitalism’ across 85 articles and book reviews. Approximating the publication of 50 articles per year over a 24-year period, less than 1% of texts in the JIEL mention capitalism. Most references are peripheral, with capitalism appearing in the title of a single book review. ‘Socialism’ surfaces in 18 texts and 0 titles, ‘communism’ in 19 texts and 0 titles, ‘colonialism’ in 28 texts and 0 titles, and ‘colonial legacies’ in 4 texts and 0 titles. Most striking, ‘racism’ appears in 8 texts and 0 titles though ‘poverty’ appears in 196 texts, while ‘democracy’ and ‘democratic’ surface in 229 and 316 texts respectively (‘plutocracy’ is used once and ‘plutocratic’ never). The journal self-describes as a site for deliberation on ‘a very broad range of subjects that concern the relation of law to international economic activity’, committed to ‘promoting peace, world welfare, and enhancement of the quality of life for all peoples.’ Notwithstanding this, capitalism and racism are beyond its radar, preserving the fiction of capitalism’s anti-racist credentials. Lang is bemused by IEL scholars for their ‘deafening silences’ (2006):^3^ ‘The story [of the trade regime and of IEL] leaves out, in Dillon’s words, “vestiges of colonialism, global racism, and a long past of global exploitation”’ (2002, p. 152).

Critical scholars, by contrast, confront vestiges and exploitative dynamics, often relying on Marx’s expose of commodity production to make them apparent (Fraser 2016; Teubner 2008, p. 330). The secret to accumulation is not much of a secret at all. Capitalism commands the exploitation of wage-labour, made possible through the inequality of distribution capitalism engenders. Lacking the means of production, capitalists leverage the human survival instinct to coerce people into toil. Since ‘workers’ cannot lay claim to any surplus value they generate, they are in a constant state of exploitation. To proclaim equivalency in the ‘exchange’ is fallacious under these circumstances, turning a blind eye to the unequal bargaining power the respective parties exploit and suffer. As Nancy Fraser remarks, capitalists compensate workers ‘only for the socially necessary cost of their own reproduction’ (2016, p. 164). This relationship validates Marx’s classification of capitalism not as an economic system of market exchange, but as a social system of class domination.

Racial capitalism scholars go further, demonstrating that class domination is shot through with race. Many scholars are relevant in this debate, and we highlight several poignant individuals throughout the article. We do not take a position on the strands of the theory—racial capitalism, race and capitalism, or racialised capitalism—and their suitability to the analysis of IEL. ‘[B]ecause race and racism are multidimensional, polyvocal, and ever changing,’ Rabaka affirms, ‘[it] requires varied kinds of criticism and conceptual counter-attacks’ (2021, p. 46). We lead with Rodney because of the links he draws between colonial political economy and capitalism, both of which explain the dialectics of racialised development and pave the way to Fraser’s theory of expropriation.

In both historical and disciplinary terms, Rodney’s work is timeless. *How Europe Underdeveloped Africa* (2018) unmasks European modernity, showcasing the predation upon which colonial-capitalist economics rest. ‘Racism, violence, and brutality’, Rodney announced, ‘were the concomitants of the capitalist system when it extended itself abroad in the early centuries of international trade’ (2018, p. 105). He does not treat development as an autonomous project, illustrated by the titles he adopts throughout his seminal text: *Africa’s Contribution to European Capitalist Development, Europe and the Roots of African Underdevelopment*, and *Colonialism as a System for Underdeveloping Africa*. Europeans did not develop by adopting discrete economic policies or making delicate regulatory decisions alone. Rather, European development resulted from interactions with non-Europeans that yielded processes of development and underdevelopment alike. Ultimately, Rodney accounted for those disadvantaged by the emergent capitalist order (Douglas 2017).

Many racial capitalism scholars built upon Rodney’s dialectics. Fraser proposes a clever analytic device—expropriation—to explain the racialised dynamics of accumulation (Fraser 2016). We employ Gathii’s work to explain Fraser’s theory of expropriation (2010). As Gathii argued, the interplay between violence and IEL runs deep. Following the 2003 invasion of Iraq, Paul Bremer, then US Presidential Envoy to Iraq, re-wrote Iraqi laws on finance, oil and gas, intellectual property, and agriculture. Bremer was unconcerned with the history, culture, or policy preferences of Iraqis. Rather, his motivation was to make the Iraqi system more amenable to transnational capital. For example, he forced Iraq to adopt profit repatriation mechanisms, while removing safeguards for local industry (Gathii 2010, p. 85). Both literally and metaphorically, the US *expropriated* Iraq of its resources, policies, and law, deploying warfare to subjugate resistance.

Another relevant manifestation involves El Banco Central de Venezuela (BCV). The BCV stores some of the national gold reserves in the Bank of England. This is standard practice for many Third World central banks (al Attar and Reay 2021). To enable patron states to access immediate liquidity when needed, they keep deposits in cities with vast financial services. In September 2018, the BCV attempted to repatriate some of its gold. The Bank of England refused to honour the request, citing the attempted coup by Juan Guiadó as evidence of uncertainty about the leadership of Venezuela, a decision the UK Court of Appeal backed (Armas 2018). Until today, the Bank of England remains steadfast. Inspired by this act of financial expropriation, the Americans followed suit. In 2022, the USA seized hard currency reserves deposited by the Central Bank of Afghanistan in the New York Federal Reserve Branch. Joseph Biden, the American President, promised to use the $7 billion of deposits, first, to benefit the Afghan people (whatever this means) and, second, to compensate a group of 9/11 victims. ‘The United States has taken steps to exploit its centrality in global capital markets to advance its foreign policy interests,’ Drezner (2022) argues.

‘[A]ccumulation requires a legal framework to guarantee property rights, enforce contracts, and adjudicate disputes,’ Fraser asserts. ‘Equally necessary are repressive forces, which suppress rebellions, maintain order, and manage dissent’ (Fraser 2016, p. 170), explaining the racialisation endemic to capitalism. Political subjection and racialisation work hand-in-hand as the inferior status imposed on racialised communities legitimises the use of economic violence against them. It operates at three levels. First, it confers ‘the status of free individuals’ on disparate groups, ensuring conflict and stratification between those legally blessed and their cursed counterparts. Next, the order expropriates certain peoples of their labour, confiscating their capacities and resources. Last, expropriation conscripts these resources into ‘capital’s circuits of self-expansion’ (Fraser 2016, p. 165). Conscription is clear in the early history of capitalism—e.g. slavery or forced labour—just as it is in its modern iteration, e.g. land grabs, predatory debt, and financial expropriation. The Venezuelan and Afghan examples verify the instrumentality of international legal rules when deployed against racialised peoples. Echoing Marx, Fraser concludes that capitalism is an ‘institutionalised social order in which racialised political subjection plays a constitutive role’ (2016, p. 166). In contrast, IEL scholars strategically exclude violence from their discipline, declaring, for example, that ‘economic warfare’ and economic sanctions lay beyond IEL (Qureshi and Ziegler 2019, p. 10).

As explained, the duality embodies the dialectics of development (Rodney 2018). Peel back the property rights and market exchanges, and we uncover a process littered with blood and bodies. Whose blood and whose bodies? Enter political subjection and the differentiation capitalism draws between ‘free subjects of exploitation and dependent subjects of expropriation’ (Fraser 2016, p. 169). The distinction is political and economic: both classifications facilitate accumulation by establishing status hierarchies. Exploited workers are ostensibly free, while expropriated workers are not. Yet, each is necessary for capitalists to achieve support for the system, pitting one class against the other (Du Bois 1935, p.532).

Free and dependent labour are symbiotic, one gives meaning and purpose to the other. The political constitution of hierarchies provokes feelings of belonging and alienation or, in Gramscian terms, consent and coercion, antagonisms that capitalists prey upon to disrupt collective resistance. Racialising tropes support these hierarchies, exploiting the vulgar marker of pigmentation to segment society: citizens and subjects, workers and servants, or whites and blacks. By codifying the status of the former, they reduce the latter to an extra-legal or, often, illegal character. Fraser refers to these labels as racialising stigmata: ‘racialisation in capitalist society appears at the point where a hierarchy of political statuses meets an amalgamation of disparate mechanisms of accumulation’ (2016, p. 172). These lines are easiest to draw when differentiating between racialised groups (Jenkins and Leroy 2021).

We find ourselves in a chicken and egg, or racialisation and racism loop. Europe and America achieved their economic and cultural ascendancy through practices of expropriation, exploitation, and dehumanisation. They carved the world into consumers, producers, and fodder, with each servicing the desires of the metropolis (Rodney 2018). Combined, they produced the racist epistemology that informs the modern order. Breaking this dynamic demands not only surmounting the disadvantages it produces for those dehumanised but curbing the privileges it occasions for its beneficiaries (Melamed and Reddy 2019). In the following section, we consider how IEL accounts for the dialectics of development.

## The Racism of International Economic Law

For the vast majority of the planet’s peoples, the global economy publicizes itself in human misery.

Cedric Robinson

Privileged actors design laws to extend the underlying epistemology’s longevity: ‘the capitalist entrepreneur creates alongside himself the industrial technician, the specialist in political economy, the organisers of a new culture, of a new legal system, etc’ (Gramsci 1971). As the scaffolding for racial capitalism, IEL is both its instrument and product, fixed by and fixated on the same distortions, organising consent to the racially capitalist order. Gramscian language captures the hegemonic character of IEL. Its role is not to explain, but to compel and, per the experiences of Black peoples, coerce when they are unconvinced. Qureshi and Ziegler declare that participants in the international legal regime ‘obey it’ for a variety of reasons, ‘ranging from consent to sanctions’ (2019, p. 14).

IEL’s disciplinary framework functions as an instrument of legitimation, manifesting biases that are both blatant and banal. For example, focusing on production rather than distribution, extolling theoretical comparative advantage while sanitising historic disadvantage, and submitting to whimsical pursuits such as endless accumulation. IEL whitewashes racial violence from its intellectual, operational, and moral universe through a mix of epistemic devices. Key to our examination is IEL’s penchant for ahistoricism, decontextualisation, and externalisation, which cements its normative boundaries and operative logics. This combination allows the ongoing exclusion of race from our understanding of economic relations. We examine each in turn.

### IEL’s Racially Capitalist Epistemic Devices

We begin with ahistoricism, reiterating a core takeaway from the previous section: orthodox IEL scholars ignore ‘vestiges of colonialism, global racism, and a long past of global exploitation’ (Dillon 2002, p. 152). Recall that across the JIEL’s nearly three decades of scholarship, we find 8 references to racism and hundreds to poverty, likely because the IFIs declare ‘poverty reduction’ as a primary goal. References to poverty are etymologically and epistemologically determinative, representing a state of want as unfortunate yet isolated, born of misfortune or moral failings. ‘The problem in Africa is not that [the British] once ruled’, boomed UK Prime Minister Johnson, ‘but that we’re not ruling anymore’ (al Attar 2021, p. 156). Not to be outdone, the former President of France, Sarkozy, declared that ‘[the] tragedy of Africa is that the African has not fully entered into history [and has] no idea of progress’ (Thomas 2012, p. 399). Sarkozy’s successor, Macron, persisted with the same canard (Ba 2017). These are not neo-fascist ramblings, but proclamations of heads of states that dictate policy at the G7, G20, and IFIs.

Mills (1997) described this exclusionary practice as the *race-ing* of space, the writing out of the polity of certain spaces as historically irrelevant when studying race. IEL scholars maintain the myth of a universal epistemology while ensuring they disjoin *race-d* spaces from its path of ‘civilisation’. ‘As the global locus of rationality’ (Mills 1997, p. 44), Europe is the only region capable of apprehending universality. ‘Such ahistoricism is required to continue the racially based systemic silencing and erasure, through the tacit perpetuation of the lack of supposed intellectual characteristics of ‘primitive’ peoples’ (Tuhiwai Smith 1999, p. 25). Ahistoricism vanishes the expropriation and dehumanisation at the origins of Euro-American development (Williams 1944) keeping its racialised dynamics apart from IEL (Linarelli et al. 2018). Hostility towards IEL’s racist history is self-evident: IEL’s legitimacy depends on the romanticism of its founding myths.

Orthodox IEL scholars exacerbate ahistoricism through practices of decontextualisation. The portrayal of poverty as a condition rather than a relationship reinforces a liberal capitalist trope, obscuring the advantages some groups gain from the impoverishment of others (Marks 2009). Notice how the methods and metrics for the study of poverty inure us to its relational character. What does it mean to live on less than $1, $1.25, or $2.00 a day? Without context or comparator, the metric collapses into itself. For example, the World Bank laments ‘pandemic-related job losses and deprivation worldwide are hitting already-poor and vulnerable people hard, while also partly changing the profile of global poverty by creating millions of “new poor”’(World Bank 2020, p. 1). While they reference ‘inclusive growth’ repeatedly, at no point do they lay out the advantages pandemic-related job losses have produced for the wealthiest actors. During the first two years of the COVID-19 pandemic, ‘[t]the world’s ten richest men more than doubled their fortunes from $700 billion to $1.5 trillion’ (Oxfam 2022). The immiseration of others remains good business (Marks 2009).

When fused with sovereignty doctrine, an absolute conception of poverty reduces it to a domestic challenge, conveniently setting aside the geopolitical character of the global economy. Linarelli et al. (2018) describe this as the inside-outside strategy, where justice, distribution, and access to essential goods are situated inside the state. Yet, IFIs assert economies are best regulated at the global level. It’s a clever tactic. To take part in the global economy, states must surrender authority over their economies to the diktats of IFIs. In exchange, IFIs will refrain from commenting on the political or moral preferences of nation-states, lest they trespass over national sovereignty. What happens outside the state is not subject to any moral demands beyond the sanctity of property, contract, and debt. Qureshi and Zeigler, mindful of this conundrum, acknowledge that ‘comparative advantage is silent as to how the benefits of economic welfare are to be shared’ (2019, p. 10). They also recognise a similar limitation in liberalisation: ‘[t]he proposition that liberalisation enhances global welfare belies […] the question: whose welfare?’ (Qureshi and Zeigler 2019, p.11). Like racial capitalism scholars, we reject abstraction, preferring to name the beneficiaries and victims of maldistribution, with the groups separated by a palpable colour line.

Externalisation rounds out our trinity of IEL’s epistemic devices. To use a facile illustration, the greatest value of gross domestic product (GDP) as a measure of economic success is its tautology, legitimising the same thinking that gives GDP meaning. As a metric, GDP presupposes that economic growth is presumptively desirable; that metrics can assess states against one another despite characteristics and endowments that batter any semblance of parity; and that states can cooperate and compete in equal measure. Much like pooling individuals of different age, background, gender, class, ethnicity, education, aspiration, etc. would produce poor comparative data, the same is true for states. We note IEL’s poverty narrative coheres with the GDP metric, enabling us to champion and celebrate growing economies even as inequality soars and life chances dwindle. As articulated by Linarelli et al. (2018), minimising state intervention polarises the distribution of resources. By externalising distribution from IEL’s frame, so too vanishes the inverse correlation between development and underdevelopment.

Biofuel production provides a potent manifestation of externalisation. ‘The increase in biofuel production contributed to the growth in commodity food prices as a result of a positive demand shock on agriculture markets’ (Subramaniam et al. 2019, p. 73). Third World states are especially vulnerable to land grabs and the redirection of agriculture policy toward servicing export markets. ‘[It] reduces food consumption in developing countries, which in turn would cause increased undernourishment’ (Subramaniam et al. 2019, p. 73). The Food and Agriculture Organisation reports similar findings: ‘When crops are used for biofuels, the first direct impact is to reduce food and feed availability. This induces an increase in prices and a reduction of food demand by the poor. It also encourages farmers to produce more’ (2013, p. 14). The World Bank concurs: ‘[C]oncerns remain about the effect of bioenergy on […] the poor people in developing countries who will be affected by the changes in land use, land tenure, and land rights it will bring about’ (2010, p. 147). What changes? ‘Most of the changes are likely to result from the planting of agricultural crops to produce ethanol and biodiesel’ (2010, p. 148). Once again, lands and lives feed the engines of industry, the racialised costs externalised in upbeat reports about biofuel production and environmental sustainability.

IEL’s racial capitalist epistemic devices extend beyond these discrete and non-exhaustive examples, manifesting in trade and investment agreements. As early as the seventeenth century, European powers adopted non-discrimination and equal treatment precepts, translated into the now irrepressible National Treatment and Most Favoured Nation clauses. We find them in the ‘Friendship Commerce and Navigation’ treaties that laid the groundwork for IEL, and FTAs, the WTO regime, and even progressive initiatives such as the AfCFTA. Yet, each clause epitomises the epistemic devices, ensuring that formal equality guides the regulation of economic interactions despite the unequal characteristics of states and the differentiated capacities they possess. Again, the ghosts of a mythical liberalism rear their heads, ensuring that we regulate all sovereign states alike. While celebrated with the same vigour as formal equality is in municipal law, the clauses expose IEL’s universal-particular dynamic. Irrespective of history, context, and impact, IEL demands states abide by identical standards.^4^ States have legitimate reasons for seeking variable forms of engagement with others that account for the particularities that shape their existence. Disabling this possibility, as the epistemic devices do, preserves the historical conditions of racial capitalism, irrespective of the racialised inequalities that result.

The perverse living conditions racialised peoples suffer today are inevitable within an IEL system that consecrates endless accumulation. We sacrifice societies for the lifestyles of wealthier states (Fuchs and Lorek 2002). IEL’s epistemic devices, normative boundaries, and operative logics suppress race from IEL’s frame. Through these devices, IEL ensures that racialisation and racism are blips on the radar, perverse rather than endemic. ‘Sympathetic connections between law and racism can be presented as exceptional and remediable, with the exceptional serving to contrast and confirm the great virtue of the norm,’ Fitzpatrick notes (1987, p. 121). Immiseration, the dialectics of development, and the penetrating language of racial capitalism scholars remove us from the sanitised spaces IEL inhabits, displaying the racialised wounds IEL conceals.

### IEL’s Normative Boundaries and Operative Logics

Equally central to our argument is a quadrant of IEL’s normative boundaries: neoclassical economics, liberalism, neoliberalism, and multilateralism. Each operates as a central assumption of IEL, naturalised in its research and teaching. As intended, each boundary limits possibilities, inculcating participants in a logic about economic and human evolution that is racialised, Eurocentric, and fantastical. We might describe these boundaries as hagiographical rather than theoretical for they negate scientific inquiry, insisting on epistemic fealty instead.

We merge each boundary with components of IEL’s operative logics: commodities, debt, distribution, and labour migration. As we argue, these assumptions relate to both the material and ideological underpinnings of racial capitalism. An enumeration, however, is misleading, as neither boundaries nor logics are exclusive or exhaustive. We aggregate and disaggregate for explanatory purposes, demonstrating these elements are decisive in demarcating IEL as a race-free and capitalism-light research zone.

### Number One: Neoclassical Economics and Commodities

Consider the supply-and-demand outlook that informs international trade law (Igwe 2018). In collaboration with neoclassical economics, the regime imposes a viewpoint that relies on abstract concepts and modelling. The logic is simple, implying that economies will grow by increasing trade with others. Sloganeering is also typical. ‘Rising tides lift all boats’, ‘strong economies benefit all people’, and ‘wealth trickles down’ are familiar refrains. Proponents of neoclassical economics account for material realities such as scarcity and sustainability within the frame itself, seeking to align these limits to the attendant logic (Schneiderman 2018). To illustrate, recall Lawrence Summers’s plea during his stint at the World Bank (Bond 2006, p. 8). He implored Third World states to leverage their comparative advantages of vast land and low population density to sell toxic waste dumping licences to First World states. Left out of the analysis is an account of the uni-directional character of some trade—e.g. waste disposal—and the imbalance in others (e.g. raw materials and finished products). By excluding inequitable outcomes, neoclassical economists theorise away the structural imbalances intrinsic to the global order and the racialised dynamics that produce and perpetuate them.

Commodities exemplify the triad of structural imbalance, racialised dynamics, and inequitable outcomes. During the colonial period, imperial powers reconstituted swathes of the (Third) world into agricultural, mineral, and human export colonies (Gardner and Roy 2020). To a staggering degree, this reality prevails for many post-colonial societies. Comparative advantage, a key theory upon which neoclassical economics and the international economic order rest, remains the guiding light in IEL, despite its obvious shortcomings (Schumacher 2013). Imperial powers shaped colonised societies into launch pads for embryonic value chains: providers of primary products and exploited labour. We need not explain the gap in added value between raw materials and finished products, and IEL doubles-down on this dynamic. Shandra et al. argue the IFIs facilitated processes of exploiting the ‘labor and natural resources of poor nations through free trade via their structural adjustment loans’ (2011, p. 213). Comparative advantage is key to this dynamic. To ‘increase export earnings in order to finance interest and principal payments,’ Third World states expand ‘production and extraction of primary products and agricultural goods for export’ (Shandra et al. 2011, p. 213). Most relevant to the dialectics of development, ‘a focus on raw material exports prevents increases in the sort of value-added industries that employ the poor’ (Shandra et al. 2011, p. 213). IEL supports increased production of raw materials, discourages investment in economic diversification, and champions market openness, all part of the zeitgeist of European liberalism and capitalism. Save for abstract references to shared prosperity, local peoples are absent from these conditions.

### Number Two: Liberalism and Labour Migration

As an ideology, liberalism beatifies liberty and autonomy transplanted to international law through its doctrines of sovereignty and self-help (Castro 2019). Both pillars are inherent in the Westphalian order. Indeed, within liberalism, it is impossible to conceive of another arrangement. Yet, this system presupposes a fixed or sedentary lifestyle that is incongruent with history and incompatible with modernity. Racial capitalism helps us appreciate that capitalism’s ‘political order is inherently geopolitical’ and that accumulation compels cross-border coordination (Fraser 2016, p. 170). In a world marked by obscene inequalities, the application of liberal logic creates conditions for the extra-legal and illegal movement of people, generating racialised patterns of mobility. The Windrush scandal that shames the UK, the refugee camps that shame the world, and the daily drownings in the Mediterranean Sea and English Channel evince the racism of borders (Achiume 2022a). Looking at the global economy, the contradictions between multilateralism and mercantilism, between movement and immobility, are clear. Still, IEL scholars insist on rationalising population flows within the IEL frame, their racialised character dismissed outright, at risk of ‘transferring and reifying existing inequalities’ (Achiume 2022b).

Achiume (2022b) argues that framing people as *labour* commodifies transnational migrants, subjecting them to determinations about national and corporate needs while discounting the exploitation and inequalities upon which their status rests. Racial capitalism enables our examination of migration flows, while accounting for ‘historical patterns of exploitation and precarity’ (Achiume and Last 2021, p. 3). Reminiscent of Fraser’s expropriated workers, ‘illegal’ migration is useful in building a precarious labour pool drawn from across borders that competes against exploited workers. The brain drain is another manifestation of peoples’ commodification. ‘The brain drain is a migration of professionals from one country to another, mainly for higher salaries and better living conditions […] to the detriment of the home countries’ (Adesote and Osunkoya 2018, p. 398). Since the 1990s, the dominant trends involved people moving from low- and middle-income countries to wealthier economies (Adesote and Osunkoya 2018, p. 400). Much research underscores the negative impact of the brain drain, with some scholars highlighting the role of IEL in encouraging this trend (Shillington 2012). Northern immigration policies targeted professionals most needed in the Third World: ‘One of the palpable consequences of brain drain is the shortage of qualified manpower [sic] in critical sectors like education, health, science, technology, and business’ (Adeyemi et al. 2018, p. 69). In the end, notional conceptions of sovereignty and self-help legitimise state-to-state competition and reward racialised migratory flows, dynamics the liberal viewpoint dismisses.

### Number Three: Neoliberalism and Debt

After two generations, we appreciate the neoliberal proposition is more than ideas and policies: at its core, neoliberalism comprises rules, regulations, institutions, and cultural precepts (Palacios Lleras 2016). While much of classical liberalism persists in world order, neoliberal thinking has substituted *laisser-faire economics* with a more interventionist outlook. To prevent economic and political power from reinforcing one another, neoliberal theorists advocate controlling economic processes that afford legalised protections for property rights (Palacios Lleras 2016). As Foucault (2008) remarked, we swapped the invisible hand for the sustaining one. Naturally, many neoliberal ideas stem from Hayek. ‘A condition of liberty in which all are allowed to use their knowledge for their purposes, restrained only by rules of just conduct of universal application’, Hayek asserted, ‘is likely to produce for them the best conditions for achieving their aims’ (as quoted in Palacios Lleras 2016, p. 64). Within neoliberal logic, the system demands authority and coercion to preserve the ‘general principles to which the community has committed itself’ (Hayek as quoted in Palacios Lleras 2016, p. 64). General principles refer to economics and the belief they provide an adequate measure for law and justice. Neoliberal legality refocuses the state toward deploying regulatory support for parochial economic activities; everything else, including social welfare, is incidental (Bhandar 2021).

However, as Mahmud argues, ‘in the neoliberal era the hidden hand of the market and the iron fist of the law work in concert to forge governmentalities that suture debt with discipline’ (2015, p. 69). While the debt crisis afflicting racialised peoples predates the 1970s, it hit stratospheric heights shortly thereafter. IFIs and private creditors directed oceans of money into the coffers of Third World capitalists (King 2016). They claimed these new loans would support the balance of payment challenges the Third World suffered. The credit line did little to redress the structural inequities that generated the challenges to begin with. Instead, as the debt burden rose, these governments became addicted to easy money and more amenable to the policy conditionalities needed to secure it. Debt is one of the more effective tools in the IEL arsenal. Inequality is inherent to the debtor transaction, operating to reinforce the coercive dynamic that perpetuates an unequal relationship (Bradshaw and Huang 1991). Not that anyone expected debt to dismantle the racial hierarchy; instead, the rising debt burden entrenched the imbalance, with the correlation between race and debt whitewashed from the discussion.

Distributional injustice naturally flows from excessive debt as opportunities available to racialised peoples narrow. With an obscene portion of national revenue earmarked for debt repayments, redistribution and reinvestment are difficult, if not impossible. Discussions about social provision in Third World states underscore the fiscal challenges and constraints they navigate, with neoliberal reforms narrowing their options (Taylor 2009). Regimes of social provision appear antithetical to a model of capitalist development. Governments structure the provisions to ensure the reproduction of the labour class and little more. The constraints are both ideological and material. Within a neoliberal paradigm, critics of social welfare doubt the efficacy of public action while celebrating markets as allocative instruments (Diller 2002). As a result, empirical evaluations of the regimes of social provision are infrequent and consistently measured against neoliberal metrics, such as efficiency, macroeconomic balance, and low tax burdens. When combined with the open economies demanded by IEL, the possibility of wealth redistribution is neutered altogether, perpetuating the imbalances between the white First World and the racialised Third World.

### Number Four: European Multilateralism and intra-African Trade Disruption

As international legal scholars increasingly acknowledge, the European multilateral system is truncated, structured on partialities that benefit the Global North at the expense of the Global South (Fagbayibo 2019). Obvious examples include voting rights at the IMF and World Bank, imbalanced dispute resolution processes at the WTO, unequal bargaining power, and a rule of law that elevates proprietary protections above other human rights. The European version of multilateralism presupposes these pillars, constructing a self-sustaining intellectual and material universe. Its hegemonic character produces not just epistemological investments, but epistemological blinds that constrain the human imaginary. We do away with other visions of world order by evaluating proposals against the dominant one, implying a presumptive legitimacy to the status quo.

To illustrate, consider the challenges facing the African Continental Free Trade Agreement (AfCFTA). The African Union and its member states have adopted the AfCFTA to advance economic integration and increase intra-African trade. Economics are not the sole aim with the preamble formalising a commitment toward improving living standards (African Union Commission 2015). Ambition notwithstanding, the AfCFTA is contentious (Akinkugbe 2020). First, Africans have been here before. It is impossible to locate a sliver of the continent that is not swathed in trade agreements: regional economic communities co-exist alongside economic partnerships, FTAs, and WTO membership. Despite this plethora of legalised integration, intra-African trade suffocates under the weight of tariffs and non-tariff barriers alike. Will another agreement hasten the economic and political harmony that eludes the continent? Second, Africa is a battleground for global economic powers. China, Europe, and the USA compete for control of the continent’s resources, trade routes, and value chains. How will they respond to efforts by African industrialists to carve out market share?

Consider the position of the EU, which, as Fagbayibo reports, talks left while negotiating right (2022, pp. 288–289). Via the European Commission, the EU lavishes praise on the AfCFTA and prospects of an inter-continental trading arrangement. Yet, the EU operates a fragmented policy toward the African continent, creating ‘negative incentive[s] for achieving trade policy coherence on the African side’ (Luke et al. 2021). European African policy is a smorgasbord, contingent on geography, development, and legal arrangement:

Integral to Europe’s patchwork of agreements and instruments is a conditionality for security of market access, particularly on non-least-developed countries, to accept and adhere to the trade regime devised and imposed by the EU for its own purposes. African countries, north and south of the Sahara, were not the demandeurs of this regime. While they have agency over the choices that are made, faced with a colonial legacy of little intra-African trade and the reality of Europe as a dominant, stable and mature market with a long history of preferential market access, the irresistible choice has been to accept the overwhelming incentive of adherence to this regime. (Luke et al. 2021)

The Commission forces African states to choose between developing regional commitments or keeping access to the European market. ‘In the sixty years of the post-colonial period, the role of Africa mainly as an exporter of commodities to the EU has remained constant,’ they assert (Luke et al. 2020, p. 1). Luke et al. concluded that the ‘EU’s trade arrangements and underlying incentives are neither pro-poor nor pro-development. At best, they are ambivalent towards African economic integration and the forward-looking Agenda 2063’ (2020, p. 202). Fagbayibo (2022) echoes their conclusion, highlighting the financial and moral dependence of the African Union on the EU, and the fragmentation this precipitated. He questions whether the programmes can lay claim to pan-Africanism while being funded in near totality by the EU; are sustainable (because of the same dependence); and are legitimate when we account for the EU’s forceful influence of policy directions.

While we cannot tackle the AfCFTA closely, it is worth reiterating that capitalism and its dynamics of expropriation and exploitation colour all trade. The success of the order requires communities capitalists can instrumentalise. For the AfCFTA, we wonder who will suffer as neoliberalism spreads across the continent. The self-constitution of a Black comprador class reinforces rather than undermines this dynamic. This class’ evolution into a full-fledged bourgeoisie, committed to the racialised logics of capitalism and white supremacy, legitimises racialised expropriation and exploitation (Kumar Baral and Chandra Karki 2020). As mentioned, capitalism cannot correct the racial violence occasioned by capitalism through liberal programmes of inclusion (Jenkins and Leroy 2021).

For IEL scholars, this is the crux of the conundrum. European colonial powers developed economic activities and the ensuing relations to racialise and commodify non-European peoples, integrating them into a nascent capitalist economy. Each epistemic device, boundary, and operative logic is fundamental to the order. The devices precipitate tendencies that are political, moral, and subjective, shaping methods and metrics that value some things while denigrating others (Perry-Kessaris 2012). While a racial capitalist lens allows us to strip back IELs racialising and racist layers, IEL’s self-articulated boundaries—in isolation from capitalism—fragment and dilute racial capitalism’s critique. Like any racial regime, IEL requires the maintenance of the illusion of epistemic totality (Robinson 2007). Epistemic closure, Bhandar tells us, ‘render[s] the system perpetually innocent and inoculate[s] the system from criticism’ (2021, p. 288). We deliberate all symptoms of unequal development whilst ignoring its catalysts. IEL is vital to this dynamic, deployed alongside development rhetoric to legitimise predation and facilitate the imposition of policies that perpetuate the unequal exchange (Melamed and Reddy 2019). To break with the dialectics of development, we must look beyond IEL for the answer to capitalism’s predation. In the following section, we consider the prospective contribution of the Black Radical Tradition.

## The (Im)Possibility of Anti-Racist International Economic Law

I’m just imagining a world where we are pushed by collaboration for the betterment of [humanity] instead of driven by competition for increased profits.

Patrice Lumumba

Proximate to the racial capitalism critique is the Black Radical Tradition (BRT), the most effective expose of epistemic racism. Gazing retrospectively and prospectively, the BRT is critical of the present while proffering visions for the future, urging reflection on the epistemological commitments that constrain us. While we cannot and should not synthesise the BRT into a single narrative, it is worth sketching its contours.

In contrast to the presumptive universality of European liberalism, the BRT begins from its subjectivity, centring ‘capitalist slavery and imperialism’ in its cosmology (Robinson 2000, p. 124). The ‘capitalist’ qualifier is key, as it amalgamates the racial exploitation that birthed a new era with the economic system that structures our current one. Its embrace of subjectivity is also significant; the BRT is neither hegemonic nor counter-hegemonic, nor nihilistic or utopian. It did not evolve in facile opposition to capitalist command, and its interlocutors do not advocate material influence within the current configuration. Rather, as Robinson argues, the BRT is ‘a revolutionary consciousness that proceed[s] from the whole historical experience of Black people’ (2000, p. 169). African culture, politics, and ontologies inspire the emancipatory thrust that guides the BRT. Propelled by the struggles of Black peoples against racial capitalism, it radiates a ‘shared sense of obligation to preserve the collective being, the ontological totality’ (Robinson 2000, p. 171). In effect, the BRT fortifies the experiences of resistance, consciousness and agency of Black peoples. As ‘living and breathing in the place blinded from view,’ the BRT enables Black peoples to situate their pasts, presents, and futures outside of the racially prejudicial parameters of white supremacy (Gordon 2001, p. xi).

According to racial capitalism, IEL devalues entire worldviews, constituting an epistemology that is culturally impenetrable yet putatively acultural. Likewise, IEL scholars expel the perspectives and theories of racialised peoples, intimating they are naïve, utopian, or illegitimate. Each mis-translation condemns unfamiliar epistemologies to the realm of fallacy. Even those who recognise systemic racism in IEL and sympathise with resistance thereto are so afflicted. For example, while acknowledging that IEL ‘can exacerbate marginalisation’, Puig dismisses a core precept of the indigeneity movement (2021, p. 2). ‘For our nations to live, capitalism must die’ - a popular refrain for many indigenous activists’ is, according to Puig, ‘rather vague’: it is ‘improbable in the short term and impossible to cross examine with more objective analysis’ (2021, pp. 2–3). We note this vision is consistent with racial capitalism and the BRT. Most indigenous activists do not seek legitimacy for their epistemologies from ‘objective analysis’ and recognise casual dismissals as a familiar form of epistemic violence. Puig’s pre-emptive restriction to European objectivity at once reaffirms and rejects racialised knowledge.

‘Hegemonizing is hard work’ (Lipsitz 1988, p. 146). The mainstream legal academy’s failure to investigate race or non-European epistemologies is not an oversight but part of a hegemonic ontology, as verified by the bleached coursebooks that punctuated this piece. Those operating within the vantage of orthodox IEL benefit from the perpetuation of a racialised status quo. It keeps things simple, consistent, and comprehensible. And while racial capitalism and the BRT present a muscular riposte, we remain sceptical of IEL’s susceptibility to transformation. As part of IEL’s ‘ontologizing and reification,’ IEL scholars treat capitalism as stillborn yet beyond death, unchanging yet always in flux (Rabaka 2016, p. 17).

However, without transformative impetus, IEL can easily fold racial capitalism into its frame: exotic and uncomfortable, but easily cheapened into a token or a totem. Like bell hooks, we worry investigations into racial difference in IEL will be ‘commodified and offered up as new dishes to enhance the white palate,’ that ‘the Other will be eaten, consumed, and forgotten’ (1992, p. 380). If we exclude racialised peoples from the conversation, lamenting their subjectivity, our efforts to make racial capitalism visible in IEL will re-inscribe the status quo. Resisting instrumentality and co-optation are key lest we, too, exploit those enduring the hard end of the system. The ‘mutual recognition of racism, its impact on both who are dominated and those who dominate’, hooks explains, ‘is the only standpoint which makes possible an encounter between races that is not based on denial and fantasy’ (1992, p. 371). We realise this starting point remains fanciful in a field that aspires to hegemonic, sometimes messianic goals.

Yet, with examples of marronage, quilombos, and runaways, the BRT confronts the improbability of a non-capitalist future. Scholars of racial capitalism do not accept the inevitability of racial capitalism, nor believe in the reform of a predatory formation. Anti-racist reforms of capitalism are conceivable only if they extend capitalist relations into non-white spaces, but fundamental change is the goal of racial capitalism and BRT scholars (Johnson and Lubin 2017). The BRT revels in the agency of peoples, extolling their alternative ways of being irrespective of subjectivity (Quan 2013, p. 121). It is in this conception of self-actualisation that the BRT draws its power (Al-Bulushi 2020). Since capitalist modernity survives by perpetuating the myth of prosperity for all, the BRT explores instances and possibilities of life outside the terms of a racially capitalist society, exploding its fallacy as the natural order (Quan 2013). What is life outside? The BRT does not provide a manual for reimagined futures. As Hall warns, we must neither ‘extrapolate a common and universal structure of racism […] outside of its specific historical location’ nor proffer contextless freedom dreams (Hall 1980, p. 337).

Contradicting a host of Third World revolutionaries, ranging from Du Bois to Bedjaoui, the BRT disavows statist conceptions of liberation. We find part of the rationale for this position in Pahuja’s quip about Third World states being born into law (Pahuja 2011, p. 153). But it goes further than this. The BRT condemns the embrace of a Eurocentric epistemology implicit in Black nationalism, pan-Africanism, and statist-capitalist models of liberation such as the New International Economic Order and the AfCFTA. If capitalism is racial and predatory, it will not metamorphose into a universal engine of liberation just because Black peoples are at the helm. ‘Perhaps the most central characteristic of the black radical tradition in Robinson’s account is that of non-sovereignty’: the BRT demands ‘non-sovereign visions of freedom’, provoking a fundamental break with the Westphalian doctrine that shaped IEL (Al-Bulushi 2020). Quan terms this a state of ungovernability: ‘those individuals and communities that render themselves unavailable for governing’ (Quan 2013, p. 132). Plainly, to break with racism requires a break with capitalism and a state-centric order.

It is here that we propose to begin a discussion about racial capitalism and the BRT’s potential contribution to IEL. By deviating from the materialism upon which capitalism rests, the BRT rejects the dismissal of Black histories, cosmologies, and epistemologies in the universe of IEL. It makes prominent the Black cultures and spiritualities that inspired Third World resistance: ‘the black radical tradition was nurtured and sustained only through its immaterial, spiritual and cultural resources’ (Al-Bulushi 2020). Foremost, the BRT begins from the position that Black peoples are human. While this seems banal today, we recall Europe spent modernity denying this basic proposition. Such denial is reflected in the development of European international law and IEL. In seeking to elaborate new visions for IEL, world order, and an anti-racist imaginary, the BRT insists upon the centrality and plurality of Black peoples and of Blackness. Where will this provocative outlook lead IEL?

Robinson forewarned ‘if we are to survive we must take nothing that is dead and choose wisely from among the dying’ (2000, p. 316). As we argue throughout this article, racism is foundational to capitalism and to IEL. To imagine an anti-racist (and anti-capitalist) IEL is to invoke its detonation. Racial capitalism’s shape-shifting, its realism-qua-pragmatism, and ever malleable epistemic framework led us to conclude that operating from within the system works to deplete the imagination needed to move beyond it. It is here that racial capitalism and the BRT trigger an epistemic rupture, allowing us to disengage with IELs existential boundaries; to embrace the freedom dreaming of Black radicalism. In doing so, the BRT’s visions of ungovernability and non-sovereignty provide a unique epistemic sphere. This will allow us to think outside the interrelationship between state, racial capitalism, and IEL in search of an alternative to order rather than an alternative to the current order (Quan 2013).

## Conclusion

If we do not know how to meaningfully talk about racism, our actions will move in misleading directions.

Angela Davis

While radicalism never depends on the intelligentsia, the *petite bourgeoisie* can take up the mantle of racial capitalism and the BRT, showing disloyalty to the epistemological contours that encircle us (Quan 2013). To reject the logic of a racially motivated epistemic loop requires recognising that our disciplinary worldview and historical moment comprise partial and anachronistic epistemological presumptions; IEL scholars, too, can pursue freedom dreaming. However, a conceptualisation of freedom that searches beyond racial capitalism and the plutocratic welfare it champions begins with racialised communities. This includes their histories, visions, theories, epistemologies, and radical traditions (Osuna 2017). Ultimately, we do not believe IEL is amenable to scholarship that challenges its white supremacy. To move beyond the discipline’s racial capitalism and white supremacy demands an upheaval of epistemic proportions. For this, we propose moving IEL’s disciplinary boundaries to not only account for but to centre the lived realities of those brutalised by the regime. Actualising an epistemologically equivalent vision is an urgent strategic undertaking, requiring a long-term vision combined with tactically informed uses of law (Knox 2010). While acknowledging the structurally racist nature of IEL, such tactical use of law creates obstacles to delimit or slow racial capitalism’s cannibalistic creep. We must pursue tactical intervention in tandem with a long-term, epistemologically revolutionary vision. What this vision is, how it will emerge, or the tactics to achieve it remain unwritten or incomplete *by design*. If racial capitalism and the BRT teach us anything, it is that struggle produces vision(s).

## Data Availability

Not applicable.

## Footnotes

1 Racial capitalism’s canon includes Oliver Cromwell Cox’s *Caste, Class, and Race*, Angela Davis’s *Women, Race, and Class*, Claudia Jones’s contributions to the *West Indian Gazette*, Kwame Nkruma’s *Neo-Colonialism: The Last Stage of Imperialism*, Cedric Robinson’s *Black Marxism: The Making of the Black Radical Tradition*, Walter Rodney’s *How Europe Underdeveloped Africa* and Edward Said’s *Culture and Imperialism*.

2 Through imperial and colonial practices, Europe collapsed the tributary system that informed pre-colonial social relations. This rupture was vital to the rise of capitalism (Amin, 2008).

3 Such silences are not unique to IEL. There is a reluctance among some scholars of race and white supremacy to reflect on the economic. Morgan notes that ‘[h]istorians understand the relationship among early modern Atlantic settler colonialism, commodity extraction, and the transatlantic slave trade, but scholarship on the relationship between money […] transatlantic slave trade as simultaneously an economic and a cultural phenomenon is rare’ (2021, p. 14).

4 Exceptions for special and differential treatment are inadequate in countering the universalising thrust, as the clauses create a reality against which we measure all agreements. As per our argument, they dominate both regulatorily and epistemologically.
